# Review of Analytical Methods and Reporting of the Polyphenol Content of Tart Cherry Supplements in Human Supplementation Studies Investigating Health and Exercise Performance Effects: Recommendations for Good Practice

**DOI:** 10.3389/fnut.2021.652094

**Published:** 2021-03-26

**Authors:** Vlad R. Sabou, Mary F. O'Leary, Ying Liu, Paula N. Brown, Susan Murch, Joanna L. Bowtell

**Affiliations:** ^1^College of Life and Environmental Sciences, Sport and Health Sciences, Exeter University, Exeter, United Kingdom; ^2^Natural Health and Food Products Research Groups, BC Institute of Technology, Burnaby, BC, Canada; ^3^Department of Chemistry, University of British Columbia, Kelowna, BC, Canada

**Keywords:** tart cherry, anthocyanins, exercise recovery, sleep, dietary supplement

## Abstract

Tart cherries (TC) are a rich source of polyphenols that elicit antioxidant and anti-inflammatory effects. As a consequence, the effects of TC derived supplements on markers of human health, exercise performance and sleep have been investigated. Supplementation protocols have been highly variable across studies and the dose of bioactive compounds used has often been poorly characterized. Specific and non-specific analytical methods were employed for measuring the total polyphenol and anthocyanin content in TC supplements. This review critically analyses the supplementation protocols and the analytical methods used for the characterization of TC supplements, culminating in recommendations for good practice in the analysis and reporting of the polyphenol content and profile of TC products. A literature search was conducted using PubMed/Medline and Web of Science up to May 4th, 2020, including studies published in all years prior. Only articles written in English that provided a TC dietary supplement as opposed to fresh whole TC were included in this review. Forty-three studies were identified as eligible and included for analysis in this review. The studies investigated the effects of TC supplementation on various aspects of human health, exercise recovery and performance and sleep. Twenty studies conducted an analysis of TC supplement and reported total polyphenol/anthocyanin content. Six studies did not report the polyphenol content of the TC supplement used. Seventeen studies reported the TC supplement polyphenol content but this was derived from previously published studies and presumably different supplement batches. The duration of the supplementation protocol ranged from acute supplementation to 84 days, meanwhile the total polyphenol and anthocyanin dose ranged from 143 to 2,140 mg/day and 15 to 547 mg/day, respectively. Due to the variety of specific and non-specific analytical methods used, the relative efficacy of different doses and polyphenol blends cannot reliably be extrapolated from critical analysis of the literature. Future studies should conduct an analysis of the study supplement batch. In addition to analysis and reporting of total polyphenol content, specific analytical methods such as HPLC UV/MS should be used to quantify total and individual anthocyanin contents.

## Introduction

Tart cherries (TC) are part of the *Prunus* species and are predominantly cultivated from the Montmorency cultivar (1). The chemical composition of TC can be affected by many parameters such as cultivar, maturation stage, agricultural practices, and environmental conditions. In general, the level of soluble solids increases as the fruit matures, whereas titratable acidity declines (2). Water and carbohydrates are the major constituents of the fruit. The main sugar in cherries is glucose with no starch present (3) and malic acid at 600–900 mg/100 grams of fresh weight is the dominant organic acid (2). All essential amino acids can be found in TC with an additional high level of melatonin in the Montmorency cultivar (3, 4). Tart cherries are considered a good source of potassium; other minerals exist in low concentrations in the fruit. There are a wide range of vitamins in TC and a noticeably high level of vitamin A. The macro- and micro-nutrient and phytochemical content of TC is compared to other common berry fruits in Table 1 (3, 5). The plant is rich in phenolic compounds which have become the focus of interest for consumers and researchers since these compounds are considered to confer their antioxidant and anti-inflammatory properties (6–10).

**Table 1:** Comparison of major nutrients, vitamins and phenolic compounds between tart cherries and other common berries.

	**Per 100 g**	**Strawberry**	**Blueberry**	**Raspberry**	**Cranberry**	**Sweet Cherry**	**Tart Cherry**
Energy^a^	kcal	32	57	52	46	63	50
Water^a^	g	91	84	86	87	82	86
Protein^a^	g	0.67	0.74	1.20	0.46	1.06	1.00
Total lipid (fat)^a^	g	0.30	0.33	0.65	0.13	0.20	0.30
Carbohydrate^a^	g	7.7	14.5	11.9	12.0	16.0	12.2
Fiber, total dietary^a^	g	2.00	2.40	6.50	3.60	2.10	1.60
Sugars^a^	g	4.89	9.96	4.42	4.27	12.82	8.49
Fatty acids, total saturated^a^	g	0.02	0.03	0.02	0.01	0.04	0.07
Potassium, K^a^	mg	153	77	151	80	222	173
Vitamin C, total ascorbic acid^a^	mg	59	10	26	14	7	10
Thiamin, B1^a^	mg	0.02	0.04	0.03	0.01	0.03	0.03
Riboflavin, B2^a^	mg	0.02	0.04	0.04	0.02	0.03	0.04
Niacin, B3^a^	mg	0.39	0.42	0.60	0.10	0.15	0.40
Pantothenic acid, B5^a^	mg	0.13	0.12	0.33	0.30	0.20	0.14
Vitamin B-6^a^	mg	0.05	0.05	0.06	0.06	0.05	0.04
Folate^a^	μg	24.0	6.0	21.0	1.0	4.0	8.0
Choline^a^	mg	5.7	6.0	12.3	5.5	6.1	6.1
Vitamin A, RAE^a^	μg	1	3	2	3	3	64
Carotene, beta^a^	μg	7	32	12	38	38	770
Vitamin A^a^	IU	12.0	54.0	33.0	63.0	64.0	1283.0
Lutein + zeaxanthin^a^	μg	26.0	80.0	136.0	91.0	85.0	85.0
Vitamin E (alpha-tocopherol)^a^	mg	0.29	0.57	0.87	1.32	0.07	0.07
Vitamin K (phylloquinone)^a^	μg	2.2	19.3	7.8	5.0	2.1	2.1
Anthocyanidins, cyanidin^b^	mg	1.68	8.46	45.77	46.43	30.21	32.57
Anthocyanidins, peonidin^b^	mg	24.85	29.29	0.12	49.16	1.50	0.87
Flavan-3-ols, (-)-epicatechin^b^	mg	0.42	0.62	3.52	4.37	5.00	3.83
Flavan-3-ols, (+)-catechin^b^	mg	3.11	5.29	1.31	0.39	4.36	0.30
Flavonols, isorhametin^b^	mg	0.00	-	0.00	-	0.05	0.72
Flavonols, kaempferol^b^	mg	0.50	1.66	0.06	0.12	0.24	0.24
Flavonols, myricetin^b^	mg	0.04	1.30	0.00	6.63	0.05	0.00
Flavonols, quercetin^b^	mg	1.11	7.67	1.05	14.84	2.29	1.47

a *(3), based on 100 g fresh weight*,

b *(5), based on 100 g edible weight, RAE, Retinol activity equivalents*.

At least 24 anthocyanins, 12 phenolic acids, 17 flavanols, and 18 flavones have been identified in tart cherries (11). Collectively this results in a high total polyphenol content, on average 352 mg of total polyphenols per 100 g of fresh weight (fw) (12) and a greatly diversified profile that includes kaempferol, quercetin, cathechins, epicathechins, proanthocyanidins and anthocyanins (13). Although the anthocyanins present in TC have received the highest degree of interest to date, it is possible that the bioactive properties of TC arise from the interaction of the various polyphenols present in this fruit, which may act synergistically in modulating various molecular pathways (14). The commonly detected anthocyanins in tart cherries are cyanidin-3-glucoside, cyanidin-3-glucoslrutinoside, cyanidin-3-rhamnoglucoside, cyanidin-3-sophorside, peonidin-3-glucoside, and peonidin-3-rutinoside (11). The types and the relative abundance of the anthocyanins vary among cultivars (15, 16).

TC polyphenols are likely to exert antioxidant and anti-inflammatory properties by modulating several cell signaling pathways (17, 18). Growing evidence indicates that rather than exerting direct antioxidant effects as radical scavengers, polyphenols upregulate endogenous antioxidant capacity via activation of the transcription factor nuclear related factor 2 (Nrf2)/antioxidant response element (ARE) pathway (19, 20). Signaling is suggested to be induced by quinones, produced via exposure of polyphenols to reactive oxygen species. *In vitro*, polyphenols have been shown to have the potential to protect the Keap1-Nrf2 complex against ubiquitylation and degradation, and to promote Nrf2 phosphorylation. Phosphorylated Nrf2 translocates to the nucleus resulting in downstream gene and protein expression [for reviews see (21, 22)], culminating with increased synthesis of phase || detoxifying and antioxidant enzymes (22). Furthermore, polyphenols may inhibit the expression and activity of superoxide producing enzymes such as nicotinamide adenine dinucleotide phosphate (NAPDH) oxidase (23–25), thereby reducing the formation of ROS. The anti-inflammatory molecular mechanisms of action for polyphenols have also been explored through *in vitro* studies. The current body of literature indicates that polyphenols inhibit the enzymatic activity and expression of the two cyclooxygenase (COX) isoforms, COX1 and COX 2 (26, 27), thus preventing the formation of prostaglandins, a group of lipid compounds that are involved in the inflammatory response by promoting swelling and pain (28). Furthermore, polyphenols may also potentially inhibit the activation of nuclear factor-κ B (NF-κB) (29), a transcription factor that modulates the expression of over 200 genes involved in the body's pro-inflammatory response. These bioactive properties provide important potential applications for TC supplementation in the management and treatment of various clinical pathologies which are linked to chronic elevation of oxidative stress and inflammation, such as cardiovascular and metabolic diseases (30–33). Indeed, there is evidence to suggest that TC supplementation is able to reduce pain and other clinical symptoms associated with knee osteoarthritis (34), to improve vascular function and cardio-metabolic markers (35–37) and to reduce uric acid markers, which has important implications for gout management (38, 39). Although, not all studies have found such favorable effects [for a review, see (40)].

Furthermore, the elevated levels of oxidative stress and inflammation identified following intense athletic competitions (7, 41, 42) contribute to the exercise-induced muscle damage (EIMD) that occurs after these events, and negatively impact exercise recovery and subsequent athletic performance. The increased production of ROS during intense exercise impairs blood flow and vasodilation (43) and may also impair calcium handling and sensitivity, and disrupt mitochondrial function (44), resulting in ergolytic effects on exercise performance. Given the antioxidant and anti-inflammatory properties of TC, the potential ergogenic effects of its supplementation for exercise recovery and performance have been investigated. To date, 8 studies showed a beneficial effect of TC supplementation on recovery following various exercise modalities including strenuous resistance-based exercise (45, 46), endurance running (7, 47) and intermittent running/cycling protocols (48–51). In contrast, seven studies found no improvements in muscle function recovery or subsequent physical performance following TC supplementation (52–58). The research on TC supplementation and exercise performance has so far been more limited and focused on endurance exercise only, with three out of four studies finding a beneficial effect of this nutritional strategy (59–61). Lastly, TC supplementation may also enhance sleep in both clinical and athletic environments (62, 63). A limited number of studies have found improvements in sleep duration and/or quality in both young (64) and older (65, 66) subjects. The melatonin content and anti-inflammatory properties of TC have been suggested to drive these beneficial effects on sleep (63, 64). Nevertheless, the mechanisms of action are yet to be established.

To date, there have been multiple narrative reviews exploring the applications of TC and derived dietary supplements for human health (40, 67), exercise recovery and performance (67–70). Furthermore, several systematic reviews have found favorable effects of TC supplementation on systolic blood pressure and systemic markers of inflammation (71), uric acid and gout (72) and endurance exercise performance (73). However, it is hard to compare the dose of bioactive compounds provided across studies since the methods utilized to analyse the composition of the TC supplements provided are highly variable. In some instances, non-specific methods such as antioxidant activity assays, total phenolic content assays, and total anthocyanin assays have been adopted. Alternatively, individual chemical constituents and specific chemical data can be generated by the use of advanced analytical instruments such as high performance liquid chromatography (HPLC), mass spectrometry (MS), and nuclear magnetic resonance (NMR). These approaches have been less commonly employed in the TC supplementation literature, although the adoption of these techniques has increased (Table 2). There is a large variability between sour cherry cultivars in terms of both total polyphenol (from 74 to 754 mg/100 g fw) and anthocyanin (21 and 285 mg/100 g fw) content and/or profile (1, 90). In addition growing conditions and post-harvest processing alter the phenolic composition, such that a wide variation in phenolic dose and blend is anticipated between supplements and studies, which will impact upon supplement efficacy. Accurate quantification and reporting of total polyphenol content and the specific polyphenol subclasses present in the TC supplements is therefore critical to determine the influence of the dose and blend of polyphenols on efficacy. None of the published reviews have attempted such an analysis due to the high degree of variability in the analytical methods used, and a reliance on the measurement of total polyphenol rather than specific anthocyanin content. In this review the TC supplementation protocols and the analytical methods used for the characterization of TC supplements are critically analyzed and best-practice recommendations made for the analysis and reporting of the polyphenol content and profile of TC products.

**Table 2:** Table presenting the extracted data from the tart cherry supplementation studies included in this review that conducted direct analysis on the dietary supplement used in the supplementation protocol.

**Study**	**Research topic**	**Tart cherry product**	**Product Volume (mL)/Weight (mg)**	**Number of times consumed per day**	**Supplementation protocol duration (days)**	**Pre-exercise loading duration (days)**	**Total polyphenol supplemented per day (mg)**	**Analytical method used for determining total polyphenol content**	**Total Anthocyanins supplemented per day (mg)**	**Analytical method used for determining total anthocyanin content**	**Study main finding**
**Health**											
Traustadóttir et al. (6)	Oxidative Stress	Tart cherry juice blend (Cherry Pharm)	236 mL	2	14	Not applicable	1,100	Spectrophotometric analysis with Folin-Ciocalteu's reagent (no reference)	119	HPLC (no reference)	Improved markers of anti-oxidant response and reduced markers of oxidative damage
Schumacher et al. (34)	Knee Osteoarthritis	Tart cherry juice(Cherry Pharm)	236 mL	2	42	Not applicable	900	Modified Folin-Ciocalteu colorimetric method (74)	60	pH differential method (75)	No difference in symptoms relief compared to placebo
Keane et al. (35)	Vascular function	Tart cherry concentrate (CherryActive)	60 mL	1	1	Not applicable	178	Modified Folin-Ciocalteu colorimetric method (76)	73.5	pH differential method (77)	Reduced systolic blood pressure
Keane et al. (78)	Phytochemical uptake following human consumption	Tart cherry concentrate (CherryActive)	30 and 60 mL	1	1	Not applicable	142.7	Modified Folin-Ciocalteu colorimetric method (76)	62.47	pH differential method (77)	Increased plasma phenolic levels following both conditions
Keane et al. (79)	Cognitive performance and vascular function	Tart cherry concentrate (CherryActive)	60 mL	1	1	Not applicable	160.75	Modified Folin-Ciocalteu colorimetric method (76)	68	pH differential method (77)	Modulated vascular function but no effect on cognitive performance
Jackman et al. (80)	Muscle protein synthesis	Tart cherry concentrate (CherryActive)	30 mL	2	14	Not applicable	Not provided	Not provided	540	HPLC (81)	No enhancement in muscle protein synthesis
Mayta-Apaza et al. (82)	Human gut microbiota	Tart cherry juice	237 mL	1	5	Not applicable	Not provided	Not provided	14.9	HPLC-DAD- ESI-Q/TOFMS (83)	Modulated gut microbiota
Martin et al. (8)	Systemic Inflammation	Tart cherry juice(R.W. Knudsen)	240 mL	1	28	Not applicable	1,827	Folin–Ciocalteu method (84)	Not measured, only indicated authenticity of phenolic profile	HPLC (85)	Reduced systemic markers of inflammation
Chai et al. (36)	Systolic blood pressure and low-density lipoprotein cholesterol	Tart cherry juice (King Orchards)	240 mL	2	84	Not applicable	450	Not provided	Not measured	Not applicable	Lowered blood pressure and LDL cholesterol
Martin and Coles (39)	Serum Urate	Tart cherry juice(Coloma Frozen Foods)	240 mL	1	28	Not applicable	993.6	Modified Folin-Ciocalteu colorimetric method (84)	15.6	Method not provided	Reduced serum urate
Lear et al. (86)	Gut microbiome	Tart cherry Concentrate	30 mL	2	28	Not applicable	1,040	Methods not provided	296	Method not provided	No change in gut microbiome, markers of inflammation or indices of glucose tolerance
Aboo Bakkar et al. (10)	Vascular function	Tart cherry powder (CherryActive)	1,700 mg	1	28	Not applicable	456	Method not reported	226	Method not reported	Attenuated vascular disfunction induced by prolonged forearm occlusion
Johnson et al. (87)	Cardiometabolic biomarkers in adults with metabolic syndrome	Tart cherry juice (Indian Summers)	240 mL	2	84	Not applicable	2,140	LC-MS (Reference not provided)	166	LC-MS	Reduced LDL cholesterol and vascular cell adhesion molecule-1. No effect on other biomarkers
**Exercise recovery**											
Connolly et al. (45)	Recovery from eccentric contractions of the elbow flexors	Tart cherry juice(Cherry Pharm)	236 mL	2	8	4	1,200	Modified Folin-Ciocalteu colorimetric method (74)	80	pH differential method (75)	Improved exercise recovery
Bowtell et al. (46)	Recovery from single-leg knee extensions	Tart cherry concentrate (CherryActive)	30 mL	2	10	7	Not provided	Method not provided	547	HPLC (81)	Improved exercise recovery
Levers et al. (53)	Recovery from lower body strength exercise	Tart cherry powder (CherryPURE)	480 mg	1	10	7	990	Method not provided	66	Method not provided	No improvement in muscle function recovery
Beals et al. (55)	Recovery from isokinetic concentric/eccentric contractions of the quadriceps	Tart cherry powder (TatVitaCherry)	30 mg	2	12	4	733	Method not provided	64	Method not provided	No improvement in muscle strength recovery and other DOMS symptoms
Lamb et al. (56)	Recovery from eccentric elbow contractions	Tart cherry concentrate (CherryActive)	30 mL	2	9	4	589	Modified Folin-Ciocalteu colorimetric method (74)	15.4	pH differential method (88)	No improvement in muscle function recovery
**Exercise performance**											
Morgan et al. (61)	15 km time-trial performance	Tart cherry capsules (CherryActive)	3 capsules	2	7	7	462.8	Folin-Denis method (no reference provided)	256.8	HPLC (no reference provided)	Improved cycling performance
**Sleep**											
Losso et al. (66)	Treatment of insomnia	Tart cherry juice (Indian Summer)	240	2	14	Not applicable	No result provided	No analysis conducted	287	HPLC (89)	Increased sleep time and sleep efficiency

## Methods

A literature search was conducted using PubMed/Medline and Web of Science up to May 4th, 2020, including studies published in all years prior. Keywords included in the search were: tart cherry, tart cherry juice, tart cherry concentrate, cardiovascular health, metabolic health, blood pressure, uric acid, gout, gut health, gut microbiome, cognitive performance, exercise recovery, exercise-induced muscle damage, exercise performance, physical performance, ergogenic effects, sleep. No data restrictions were placed for publication date or subjects age, however only articles written in English were eligible for inclusion in this review. Only studies that used a tart cherry dietary supplement (concentrate, juice, powder, capsules or gels) as opposed to fresh whole tart cherry were eligible for this review. Study protocols, abstracts of conference proceedings, animal and *in vitro* studies were excluded. The forward citation and the reference lists of the articles identified to be eligible for this review have also been screened for additional eligible studies.

**Figure 1:**
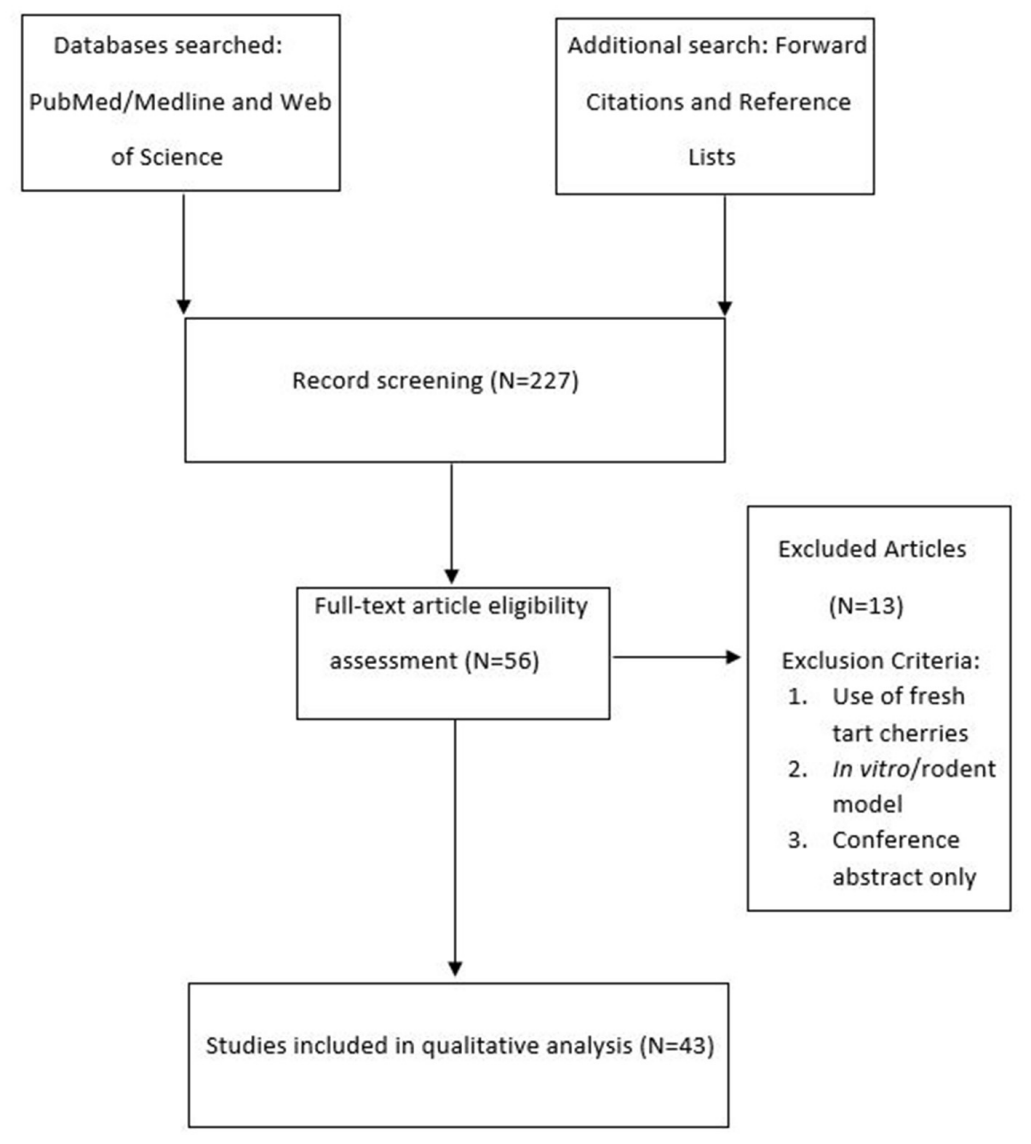
Diagram illustrating the literature search. The number in brackets indicates the total number of studies identified.

A total number of 43 studies were found eligible for this review (Figure 1), ranging in their topic from the application of TC supplementation for various aspects of human health, to exercise recovery, exercise performance and sleep. The following data were extracted from the identified studies: location, topic, participants characteristics, dietary supplement used, dietary supplement volume/weight, number of times the supplement was consumed per day, dietary supplement manufacturer, duration of the supplementation protocol, supplementation protocol pre-exercise loading (where applicable), total polyphenol and anthocyanin dosage per day, analytical methods used for measurement of total polyphenol and anthocyanin content and main study findings.

## Results

Extracted data were grouped based on the analytical methods used for TC analysis and presented in three tables. Table 2 includes the studies where direct primary analysis of total polyphenol and anthocyanin content (20/43) was conducted. Table 3 describes studies where no quantification of total polyphenol and anthocyanin content was performed, but values from previously published articles were reported (17/43). Table 4 describes studies that have reported no total polyphenol and anthocyanin content values (6/43).

**Table 3:** Table presenting the extracted data from the tart cherry supplementation studies included in this review that reported the characterization (polyphenol and anthocyanin content) from previously published studies.

**Study**	**Research topic**	**Tart cherry product**	**Product Volume (mL)/Weight (mg)**	**Number of times consumed per day**	**Supplementation protocol duration (days)**	**Pre-exercise loading duration (days)**	**Total polyphenol reported per day (mg)**	**Total Anthocyanins reported per day (mg)**	**Article used for polyphenol content reporting**	**Study main finding**
**Health**										
Bell et al. (38)	Uric acid concentration	Tart cherry concentrate (CherryActive)	30 mL	1 or 2	2	Not applicable	Not measured	547	Bowtell et al. (46)	Decrease in serum urate and increase in urinary urate excretion
Lynn et al. (91)	Arterial Stiffness and Inflammation	Tart cherry concentrate (CherryActive)	30 mL	1	42	Not applicable	Not measure	547	Bowtell et al. (46)	No improvement in arterial stiffness or markers of inflammation
Desai et al. (92)	Fat oxidation during exercise and cardio-metabolic markers	Tart cherry concentrate (CherryActive)	30 mL	2	20	Not applicable	Not measured	547	Bowtell et al. (46)	No effect of fat utilization during exercise or cardio-metabolic markers
Chai et al. (9)	Biomarkers of Inflammation and Oxidatve Stress	Tart cherry juice (King Orchards)	240 mL	2	84	Not applicable	450.6	Not measured	Chai et al. (36)	Reduced markers of inflammation and oxidative stress
Chai et al. (93)	Cognitive performance	Tart cherry juice (King Orchards)	240 mL	2	84	Not applicable	450.6	Not measured	Chai et al. (36)	Improved aspects of cognitive performance
Desai et al. (37)	Cardio-metabolic markers	Tart cherry concentrate and tart cherry capsules (CherryActive)	30 mL 4,350 mg	1	1	Not applicable	Not measured	270	Bowtell et al. (46)	Reduced insulin concentration and systolic blood pressure
**Exercise recovery**										
Kuehl et al. (47)	Muscle pain following long-distance running	Tart cherry juice blend (CherryInc)	350 mL	2	8	7	1,200	80	Connolly et al. (45)	Reduced subjective muscle pain
Howatson et al. (7)	Recovery following marathon running	Tart cherry juice (CherryPharm)	236 mL	2	8	5	1,200	80	Connolly et al. (45)	Improved muscle function recovery
Bell et al. (52)	Recovery from repeated days of high-intensity stochastic cycling	Tart cherry concentrate (CherryActive)	30 mL	2	7	4	Not measured	547	Bowtell et al. (46)	No improvement in subsequent performance. Reduction in systemic markers of oxidative stress and inflammation
Bell et al. (48)	Recovery from high-intensity cycling	Tart cherry concentrate (CherryActive)	30 mL	2	8	4	Not measured	547	Bowtell et al. (46)	Improved muscle function recovery
Bell et al. (49)	Recovery from intermittent running	Tart cherry concentrate (CherryActive)	30 mL	2	7	4	178	73.5	Keane et al. (35)	Improved muscle function recovery
McCormick et al. (54)	Recovery from water polo specific training	Tart cherry concentrate (CherryActive)	30 mL	3	6	6	Not reported	810	Bowtell et al. (46)	No improvement in recovery and next day performance
Brown et al. (50)	Recovery from an intermittent sprinting protocol	Tart cherry concentrate (CherryActive)	30 mL	2	8	4	178	73.5	Keane et al. (35)	Improved muscle function recovery
Morehen et al. (94)	Recovery following a professional rugby match	Tart cherry concentrate (CherryActive)	30 mL	2	7	5	Not reported	640	Manufacturer	No improvement in muscle function recovery or markers of inflammation
**Exercise performance**										
Levers et al. (59)	Endurance exercise performance	Tart cherry powder (CherryPURE)	480 mg	1	10	7	990	66	Levers et al. (53)	Faster half-marathon timing in the tart cherry group
**Sleep**										
Pigeon et al. (65)	Sleep duration and quality in subjects suffering of insomnia	Tart cherry juice blend (CherryPharm)	236 mL	2	14	Not applicable	1,100	119	Traustadóttir et al. (6)	Decrease in minutes awake after sleeponset
Howatson et al. (64)	Sleep duration and quality in healthy subjects	Tart cherry concentrate (CherryActive)	30 mL	2	7	Not applicable	Not measured	547	Bowtell et al. (46)	Improvement in sleep duration and quality

**Table 4:** Table presenting the extracted data from the tart cherry supplementation studies included in this review that did not include any characterization of the dietary supplement used.

**Study**	**Research topic**	**Tart cherry product**	**Product Volume (mL)/Weight (mg)**	**Number of times consumed per day**	**Supplementation protocol duration (days)**	**Pre-exercise loading duration (days)**	**Study main finding**
**Health**							
Stamp et al. (95)	Serum urate and frequency of gout flares	Tart cherry concentrate (Cherryvite)	7.5, 15, 22.5, 30 mL	2	28	N/A	No decrease in serum urate or frequency of gout flares
**Exercise recovery**							
Kupusarevic et al. (58)	Recovery following a professional rugby union match	Tart cherry gel (Healthspan)	30 mL	2	5	2	No improvements in muscle soreness or daily well-being scores
Abbott et al. (57)	Recovery following a professional football match	Tart cherry gel (Healthspan)	30 mL	2	3	0	No improvement in muscle function recovery
Quinlan and Hill (51)	Recovery following intermittent running	Tart cherry concentrate (Holland and Barrett)	30 mL	2	8	5	Improved muscle function recovery
**Exercise performance**							
Keane et al. (60)	Cycling performance	Tart cherry concentrate (CherryActive)	60 mL	1	1	1	Improved some aspects of cycling performance
Davis and Bellar (96)	Cycling performance	Tart cherry powder (Anderson Global Group)	500 mg	1	7	7	No effects on cycling performance or muscle oxygenation

Twenty studies that report conducting an analysis of TC supplement total polyphenol/anthocyanin content are included in Table 2. A large variation was identified in the supplementation protocols used within these studies. The duration of the supplementation protocols ranged from acute supplementation where only one TC dose was provided (35, 78, 79), to 84 days of chronic supplementation (9, 93). The type of dietary supplements were TC juice, concentrate, powder and capsules, produced by various manufacturers. Out of the 20 studies, 16 studies measured the total polyphenol content of the dietary supplement provided, meanwhile the anthocyanin content was measured and indicated in 18 studies. In total, 14 of these 20 studies indicated both the total polyphenol and anthocyanin content of the dietary supplement used. In the studies that conducted direct analysis of the supplement used in the study, the total polyphenol dose supplemented by participants ranged from 143 to 2,140 mg/day, meanwhile the anthocyanin dose ranged from 15 to 547 mg/day. The analytical methods used for determining the total polyphenol content of dietary supplements include several modified colorimetric methods based on the Folin-Ciocalteu or the Folin-Denis reagent and a liquid chromatography-mass spectrometry method, meanwhile several studies did not indicate the analytical method used, despite reporting that an analysis was conducted. The analytical methods used for determining the anthocyanin content included several HPLC-based and pH differential methods. Similarly, several studies provided the anthocyanin content of the dietary supplement used but did not indicate the analytical method used for this measurement.

Seventeen studies are presented in Table 3. Although values for the total polyphenol and/or the anthocyanin content of the dietary supplement used are reported in the studies included in this table, these values were reproduced from previously published studies. The duration of the supplementation protocols ranged from acute supplementation where only one TC dose was served (37), to chronic supplementation with a duration of up to 84 days (36). The type of dietary supplements used were TC juice, concentrate, powder and capsules, produced by various manufacturers. The reported total polyphenol and anthocyanin dose supplemented by participants ranged from 178 to 1,200 mg/day and 66 to 810 mg/day, respectively.

Six studies were included in Table 4. The duration of the supplementation protocol ranged from acute supplementation where only one TC dose was provided (60) to 28 days of supplementation with two daily doses (95). The type of dietary supplements used in these studies were TC concentrate, gel and powder. No values for the total polyphenol or the anthocyanin content of the dietary supplements used were reported in these studies.

## Discussion

The goal of this review was to critically analyse the analytical methods employed to characterize TC supplement phenolic composition with the aim of generating evidence-based recommendations for good practice in the analysis and reporting of the polyphenol content and profile of TC products in future studies.

From the studies included in this review, less than half conducted an analysis of the specific batch of TC supplement used, with an even lower number (~30%) reporting both the total phenolic and anthocyanin content. Furthermore, specific and non-specific analytical methods were used in these studies for the characterization of TC supplements, with the latter being most frequently utilized. Total phenolic content assays, such as Folin-Ciocalteu, and total anthocyanin content assays based on the pH differential methods are common non-specific methods used by researchers for estimating sample phenolics (97–100). These analytical methods were also used frequently in the TC supplementation literature (34, 35, 45, 56, 78, 79). Furthermore, although less frequently encountered in the studies identified in this review, other non-specific analytical methods, for example antioxidant activity measurements, inducing oxygen radical absorbance capacity (ORAC), DDPH, and ABTS, have been previously used for examining the chemistry of TC (101, 102). Nevertheless, in recent years, researchers have come to recognize the limitations of these non-specific methods, as they are susceptible to pH, solvent, and sample matric effects (98, 99, 103). Methods such as Folin-Ciocaleteu, ABTS and ORAC are based on the reductive capacity of the compounds. However, plant extracts contain a wide range of chemicals, many of which exhibit reductive capacity. Especially for fruits, like berries and oranges, strong interferences can be found with components, such as organic acids and reducing sugars, which greatly undermine the reliability of the results (100). It has been long acknowledged that there is a lack of agreement between data obtained from the HPLC methods and the Folin-Ciocaleteu method (85). The Folin-Ciocalteu assay appeared to under-report total phenolic concentration and displayed no significant correlation with total phenolics via HPLC (104). Total anthocyanin content assays based on pH differential methods work under the assumption that all aglycones in solution are the hydrolysis products of anthocyanins, despite the fact that other compounds may have the same aglycone once hydrolysed (105). A comparative study between pH differential and HPLC methods for total anthocyanin contents in tart cherry juice revealed that the difference in data between the two methods can be over 2-fold (106). Even though there was a good correlation between the two methods, the pH differential method tends to underestimate the total anthocyanin content (106–108). This under reporting has also been found in method-comparison studies of other berries and was demonstrated to be a result of the difference in glycone in the berries (108). The types of glycone vary based on berry types and the impact of such variations on the pH differential method data is observed but not well-controlled or understood (108). In addition to this lack of agreement between total anthocyanin measurement methods, a simple total phenolic value not do justice to the complexity of the chemical profile of the plant. Very little chemical information can be obtained from the value. For example, there was little to no difference in the total phenolic content between green cabbage and pear (58 mg vs. 60 GAE/100 g fresh weight), even though their individual phenolic profiles differ substantially (109).

The analysis of anthocyanins is complicated because of their ability to undergo structural transformations, which makes them highly reactive and easily degraded during storage and analysis (110). Recent advances in chromatographic methods, such as HPLC UV/MS, have enabled researchers to provide more specific and accurate quantification of anthocyanin in tart cherries. Individual anthocyanin in tart cherries has been identified and quantified. Such methods are often tailored to the target anthocyanin and sample matrix, with optimized sample preparation and analysis conditions that minimize anthocyanin degradation and transformation. Liquid chromatography has the capability of determining individual anthocyanin levels to mg/kg or μg/kg, depending on the detection method. HPLC was used in a number of studies identified in this review for measuring the total anthocyanin content and the main anthocyanin groups present in TC supplements (6, 46, 61, 66, 80). The use of these analytical methods instead of the pH differential methods represents an important step toward identifying the role played by the individual anthocyanin families in the efficacy of TC supplements. Furthermore, using HPLC UV/MS for measuring the other flavonoids and polyphenol groups present in TC supplements would provide further insight into the importance of the polyphenol profile for various health and exercise related goals. The major challenge of using HPLC, especially HPLC UV, for quantifying individual anthocyanin is the difficulty in obtaining the anthocyanin reference standards. The estimated number of anthocyanins found in nature are over 550 (111); however, only 5% of these are commercially available as reference standards (112). Thus, HPLC coupled to various type of MS is preferable since these approaches allow exact molecular weight determinations (113). HPLC-MS is often used for determination of anthocyanin in TC juice (87), due to the low level of anthocyanin this dilute sample (e.g., vs concentrate); hence there is a need for a more sensitive MS detector. However outside of juice analyses, this HPLC-MS approach is less often encountered compared to HPLC UV. This is due to its high cost and technical difficulty.

Nevertheless, from the studies that did employ HPLC (Table 2), only three studies included a detailed HPLC method. The remaining either provided no information beyond stating that an HPLC method was used or indicated that a third-party company completed a HPLC analysis. Although a published method was used by the third-party company (81) with some method validation information such as recovery and precision, neither the detection limit nor the reproducibility of the method was provided. Method validation is a process of using experimental design to prove that the method fits its intended purposes and produces accurate and precise results, all of which are fundamental to the analysis (114). Evaluating parameters, such as specificity, precision, linearity, accuracy, range, detection limit, quantitation limit, robustness, and system suitability, is a basic requirement of the United States Pharmacopeia and AOAC INTERNATIONAL for analytical methods (114, 115). Inadequate validation or lack of validation, especially transferring methods between sample types, such as from grape juice to tart cherry (8), can compromise the reliability of the data. Tart cherry showed a unique chemical profile and matrix effects in the literature, indicating a strong need for a method validation process to ensure the accuracy of the data. Furthermore, to date, no studies investigating TC supplementation effects on human physiology have used HPLC-MS or HPLC UV for measuring the other flavonoids present in the supplement in addition to the anthocyanin content. LC-MS and UV-visible MS were however previously used for determining the total polyphenol content and the amount of individual polyphenolics, respectively, present in a New Zealand blackcurrant juice (116). A similar approach could be applied for an optimal characterization of TC supplements.

In the studies where direct analysis of the supplement was conducted (Table 2), a large variation in the total polyphenol and the anthocyanin content of the TC supplements used, and thus in the daily supplemented dose, were found. These large differences in the quantity of total polyphenols and anthocyanins supplemented are driven by the different types of TC supplement used (different TC quantity & concentration used for supplement production), the natural variation in the polyphenol content and profile of TC and the different intended daily doses of supplement provided. The different analytical methods used for the characterization of TC supplements also potentially influence the findings. Large differences in the anthocyanin content of TC supplements appear to exist [between the studies that have used HPLC analysis [60 to 273 mg/30 mL; (6, 46, 66, 80)] and those that used various pH differential methods [7.5 to 40 mg/30 mL; (34, 35, 45, 56, 78, 79)]. Furthermore, differences in the total phenolic content of the TC supplements also tend to exist between the studies that used different versions of the Folin-Ciocalteu method, with the modified version based on Shahidi (76), consistently showing lower values (35, 78, 79) compared to the values identified in the studies (34, 39, 45, 56) that used previous versions of the Folin-Ciocalteu method [(74) or (84)]. These data highlight the importance of standardizing the analytical methods used for the characterization of TC supplements, with the goal of allowing effective comparisons among the studies. Ultimately, this would represent an important step toward the development of optimal total polyphenol dose and polyphenol profile recommendations for TC supplementation based on critical analysis of published studies. At present the degree of variability in the methods employed preclude such an approach.

Of the studies included in this review, 40% reported TC supplement phenolic values derived from previously published studies for the same supplement type. An additional study (94) indicated the total anthocyanin content provided by the manufacturer, without batch-testing the TC supplement used. Given the high natural variation in the total polyphenol content and polyphenol profile of TC (1, 90) the values provided in the studies above may not be an accurate representation of the phenolic composition of the consumed TC supplements. As a consequence, any comparison of dose and polyphenol blend across studies to draw conclusions regarding the efficacy and optimal TC polyphenol dose and blend is flawed. Several additional studies did not provide any characterization of the dietary supplements used. These studies compound the lack of clarity surrounding the polyphenol dose and profile provided via TC supplementation.

Alongside these analytical shortcomings within the TC literature, it is important to briefly note that the supplementation protocols used within the studies included in this review also varied considerably. Large differences were identified between studies with regard to both the duration of the supplementation protocol and the intended daily polyphenol and anthocyanin dose provided. Further, there is considerable variability in the type of TC supplements used across the studies. When these sources of heterogeneity are considered in parallel with the inadequate analytical methods employed for characterizing TC supplements, comparisons across research studies are not reliable. There is no standard reference material or consistent sample that could provide a reliable measure for comparison between studies. This raises a substantial challenge to the critical analysis of the literature in order to derive evidence-based recommendations for TC supplementation protocols.

### Recommendations for Tart Cherry Supplement Polyphenolic Analysis

In order to allow for greater confidence in future crossstudy comparisons, better data on supplement composition is required. On the basis of the existing discrepancies in the literature, we recommend that future TC supplementation studies should; (a) use specific analytical methods such as HPLC UV/MS to quantify total and individual anthocyanin content and to characterize other polyphenol contents, (b) provide an analysis of the study supplement batch and appropriate controls, (c) provide multiple timepoint analyses to demonstrate stability of the supplement polyphenol content throughout the study period and (d) provide quantitative data for all of the different formulation types used in the study. A repository of frozen supplement samples could be a useful tool for cross study comparisons. Indeed, samples could be shared between labs for standardization purposes. In the absence of certified reference materials for TC supplements, a standard supplement preparation included with each analytical batch could serve as a measure of method stability, accuracy and precision. Adoption of these recommendations in future studies would allow comparison of polyphenol dose across studies and allow researchers and applied nutrition practitioners to generate evidence-based recommendations for TC supplementation strategies.

## Conclusion

In conclusion, there is a high degree of variation in the supplementation protocols and the analytical methods used within the TC supplementation literature. Over 50% of the studies conducted to date reported polyphenol and anthocyanin content of the TC supplement from previously conducted studies rather than the specific study batch, or did not provide any characterization of the supplement used. Where direct analyses were conducted, both specific and non-specific methods were used. However, non-specific assays for measuring total phenolics content and total anthocyanin content predominate and these do not fully reflect the complex chemistry of TC supplements. Using these approaches alone to draw conclusions regarding the relationship between specific chemicals or classes of chemicals and health or performance outcomes is not appropriate. Specific analytical methods are required to better understand how the chemical nature of plants and ultimately how consumption of those chemicals may influence health, exercise and sleep.

## Author Contributions

VS was responsible for selecting the studies eligible for this review and for extracting the required data for subsequent analysis. All authors were involved in manuscript writing (review and editing), study design and methodology development and agree to be accountable for the content of the work.

## Conflict of Interest

The authors declare that the research was conducted in the absence of any commercial or financial relationships that could be construed as a potential conflict of interest.
